# Efficacy monitoring using amyloid and tau PET imaging in Down syndrome populations

**DOI:** 10.1002/alz.094705

**Published:** 2025-01-09

**Authors:** Bradley T. Christian, Matthew D Zammit

**Affiliations:** ^1^ Waisman Center, University of Wisconsin‐Madison, Madison, WI USA; ^2^ Alzheimer’s Biomarker Consortium ‐ Down Syndrome (ABC‐DS), Madison, WI USA; ^3^ Alzheimer’s Disease Research Center, University of Wisconsin‐Madison School of Medicine and Public Health, Madison, WI USA

## Abstract

**Background:**

The triplication of the chromosome containing the gene for APP production conveys a very high risk for people with Down syndrome to develop Alzheimer’s disease by their fifth decade of life.

**Method:**

The Alzheimer’s Biomarker Consortium ‐ Down Syndrome study has tracked the trajectory of PET‐measured amyloid and tau burden in the brains of individuals with Down Syndrome (>25 yrs of age) to carefully characterize their relationship with AD‐related cognitive decline.

**Result:**

Compared to sporadic AD in the neurotypical population, there is a carefully characterized accelerated time course between the emergence of these neuropathologies and the presentation of MCI and clinical dementia in the Down syndrome population.

**Conclusion:**

Clinical drug trials for the treatment and prevention of AD in Down syndrome are now being launched that use these biomarker data to inform and optimize study design. This presentation will discuss the time course of amyloid and tau within the context of treatment strategies for the removal or prevention of accumulation of AD neuropathology in Down syndrome.

